# Impact of the COVID-19 pandemic on the self-care and health condition of the older adults. CUIDAMOS+75. A mixed methods study protocol

**DOI:** 10.3389/fpubh.2024.1389641

**Published:** 2024-06-17

**Authors:** Milagros Rico-Blazquez, Silvia Esteban-Sepúlveda, Raquel Sánchez-Ruano, Ana María Aritztegui-Echenique, Eva María Artigues-Barbera, Pedro Ruymán Brito-Brito, Elvira Casado-Ramirez, María Ángeles Cidoncha-Moreno, María Inmaculada Fabregat-Julve, Isabel Feria-Raposo, Montserrat Hernandez-Pascual, Cristina Lozano-Hernández, María Teresa Moreno-Casbas, Pedro Otones-Reyes, Ana María Palmar-Santos, Azucena Pedraz-Marcos, Esperanza María Romero-Rodriguez, María Cristina Solé-Agustí, Joana María Taltavull-Aparicio, María Clara Vidal-Thomas, Víctor Manuel Gonzalez-Chorda, Cuidamos+75 Group, Rico Milagros

**Affiliations:** ^1^Research Unit, Primary Care Assistance Management, Madrid Health Service, Madrid, Spain; ^2^Research Network on Chronicity, Primary Care and Health Promotion -RICAPPS-(RICORS), ISCIII, Madrid, Spain; ^3^Gregorio Marañón Health Research Institute, Madrid Health Service, Madrid, Spain; ^4^Departament d'Infermeria Fonamental i Medicoquirúrgica, Escola d'Infermeria, Facultat de Medicina i Ciències de la Salut, Universitat de Barcelona (UB), L'Hospitalet de Llobregat, Barcelona, Spain; ^5^Institut d'Investigació Biomèdica de Bellvitge (IDIBELL), L'Hospitalet de Llobregat, Barcelona, Spain; ^6^Biosanitary Research and Innovation Foundation of Primary Care (FIIBAP), Madrid, Spain; ^7^Subdirección de Cuidados, Atención Domiciliaria, Sociosanitaria y Acciones Comunitarias, Gerencia de Atención Primaria de Navarra, Servicio de Navarro de Salud – Osasunbidea, Navarra, Spain; ^8^Facultad de Ciencias de la Salud, Universidad Pública de Navarra, Navarra, Spain; ^9^Balàfia Primary Care Center, Gerència Territorial Lleida, Institut Català de la Salut (ICS), Lleida, Spain; ^10^Fundació Institut Universitari per a la Recerca a l'Atenció Primària de Salut Jordi Gol i Gurina (IDIAPJGol), Barcelona, Spain; ^11^Multidisciplinary Research Group in Therapeutics and Interventions in Primary Care (RETICAP), IDIAPJGol, Barcelona, Spain; ^12^Department of Nursing, University of la Laguna, Santa Cruz de Tenerife, Spain; ^13^Training and Research in Care, Primary Care Management Board of Tenerife, The Canary Islands Health Service, Santa Cruz de Tenerife, Spain; ^14^Nursing and Healthcare Research Unit (Investén-isciii), Instituto de Salud Carlos III, Madrid, Spain; ^15^Frailty and Healthy Ageing-CIBERFES, Biomedical Research Center Network for Frailty and Healthy Ageing (CIBERFES), Instituto de Salud Carlos III, Madrid, Spain; ^16^IIS Bioaraba, General Head Office of Osakidetza, Basque Health Service, Subdirection of Nursing, Vitoria-Gasteiz, Spain; ^17^Primary Care Nursing Department, Castellón Health Department, Castelló de la Plana, Spain; ^18^Nursing Department, Universitat Jaume I, Castelló de la Plana, Spain; ^19^Benito Menni CASM, Barcelona, Spain; ^20^FIDMAG Research Foundation, Barcelona, Spain; ^21^Networked Biomedical Research Center, Mental Health (CIBERSAM), Barcelona, Spain; ^22^Technology and Humanization Projects Service Manager, General Directorate of Humanization and Patient Care, Ministry of Health of the Madrid Regional Government, Madrid, Spain; ^23^San Andrés Healthcare Center, Primary Care Assistance Management, Madrid Health Service, Madrid, Spain; ^24^Nursing Department, Faculty of Medicine, Universidad Autónoma de Madrid, Madrid, Spain; ^25^Puerta de Hierro-Segovia de Arana Health Research Institute, Madrid Health Service, Madrid, Spain; ^26^Maimonides Biomedical Research Institute of Cordoba (IMIBIC), Reina Sofia University Hospital, University of Cordoba, Cordoba, Spain; ^27^Nursing Department, Area 1, Murcia-oeste, Murcia Health Service (SNS), Murcia, Spain; ^28^Primary Care Research Unit of Mallorca (IB-Salut), Balearic Health Service, Palma, Spain; ^29^Research Group in Primary Care and Promotion-Balearic Islands Community (GRAPP-caIB), Health Research Institute of the Balearic Islands (IdISBa), Palma, Spain; ^30^Nursing Research Group (Code 241), Universitat, Jaume I.Castellón, Castelló de la Plana, Spain

**Keywords:** aged, big data, caregivers, COVID-19, nursing, nursing diagnosis, primary health care, mix method design

## Abstract

**Aims:**

To assess the impact of the COVID-19 pandemic on the health condition of people ≥75 years of age and on their family caregivers in Spain.

**Design:**

Multicentric, mixed method concurrent study.

**Methods:**

This work, which will be conducted within the primary care setting in 11 administrative regions of Spain, will include three coordinated studies with different methodologies. The first is a population-based cohort study that will use real-life data to analyze the rates and evolution of health needs, care provision, and services utilization before, during, and after the pandemic. The second is a prospective cohort study with 18 months of follow-up that will evaluate the impact of COVID-19 disease on mortality, frailty, functional and cognitive capacity, and quality of life of the participants. Finally, the third will be a qualitative study with a critical social approach to understand and interpret the social, political, and economic dimensions associated with the use of health services during the pandemic. We have followed the SPIRIT Checklist to address trial protocol and related documents. This research is being funded by the Instituto de Salud Carlos III since 2021 and was approved by its ethics committee (June 2022).

**Discussion:**

The study findings will reveal the long-term impact of the COVID-19 pandemic on the older adults and their caregivers. This information will serve policymakers to adapt health policies to the needs of this population in situations of maximum stress, such as that produced by the COVID-19 pandemic.

**Trial Registration:**

Identifier: NCT05249868 [ClinicalTrials.gov].

## 1 Introduction

The coronavirus disease 2019 (COVID-19) has caused a significant social and economic impact worldwide since the appearance of the first cases in the city of Wuhan (China) (1). The pandemic forced public health authorities to impose drastic measures as an epidemiological containment strategy. The Spanish government decreed a state of alarm across the national territory on March 14, 2020, which limited the free movement of citizens to essential acts and ordered home confinement for the population. In other countries, in addition to these measures, public transportation was shut down (2). Ninety-eight days later, on June 21, the state of alarm came to an end.

On a worldwide level, containment measures to reduce disease transmission were effective, especially home confinement (3), although they also entailed a substantial social impact (4) and a negative effect on physical activity levels, sleep quality, and wellbeing (5). These unprecedented measures, along with the rapid and unpredictable evolution of the pandemic, caused natural responses of fear, stress, anxiety, and loneliness among the population (6). Furthermore, the self-care and health management in people suffering from multiple comorbidities and preexisting mental disorders were adversely affected by the subsequent difficulties in accessing social-health services (7).

Numerous studies have analyzed the impact of the COVID-19 pandemic and the adopted preventive measures on the population. The social-distancing regulations imposed in all countries to control the pandemic have brought significant psychosocial consequences for individuals (8, 9).

This impact has been especially noteworthy in vulnerable groups such as children, migrants, people at risk of exclusion, individuals with psychiatric pathology, the older adults, and caregivers (8). The lock-down has been specially difficult to the older adults people, in terms of shielding and self-isolation. The lack of social communication and community networking could have affected the older adults's mental health, as well as the engagement with their own health needs and management (10).

Additionally, the technological gap experienced by these groups has aggravated this effect, increasing their anxiety levels and sense of abandonment (11). A qualitative study (12) reported repercussions on the emotional experience, coping resources, and the information received, as well as difficulties in self-care and access to health services for this vulnerable population, all while suggesting lessons to be learned for the future.

Social support is one aspect that may have influenced these results, as it is a natural tool for coping with traumatic situations and a good predictor of the resilience that a person will exhibit after a stressful experience (13). Nevertheless, support networks have not been sufficiently studied, despite their relevance for the older adults or dependents throughout the pandemic (14). Loneliness and isolation are associated with reduced longevity and worse health perception (15). Increases in sedentary activities and reductions in physical activity (16) can exacerbate frailty, and dependency for basic activities of daily living (17).

During the first weeks of the pandemic, the Spanish health system was overwhelmed by the high number of cases, which led to a reorganization and reorientation of health services in the following months according to the European Union recomendations. The growing needs of the vulnerable population during the pandemic, together with the fact that professional care was reduced, implied an overload of care for family caregivers. These were often forced to reorganize the family and care structure (17), with the added difficulty that it was not always possible for them to access the dependent's home for care (9).

In light of the above, characterizing the change in care needs of this population and the impact on their utilization of services is warranted. Standardized nursing languages (SNLs) are powerful descriptors of conditions requiring care (18). SNLs also allow for identifying situations of dependence and vulnerability, as well as describing care needs on a large scale (19). Specifically, nursing diagnoses recorded in electronic medical records (EMRs) show good predictive value to use social and healthcare resources (18).

Analyzing the utilization of health services through the evolution of SNLs in EMRs in conjunction with a mixed-methods approach (20) will provide insights into the medium- to long-term health impact, how the reorientation of services has occurred, and the experiences of the older adults and their family caregivers.

## 2 Methods

### 2.1 Aims

The main aim is to assess the impact of the COVID-19 pandemic on the health condition of people aged ≥75 years in Spain. Specifically, this study aims at:

1. Analyzing the evolution of the rates of needs, provision of care, and utilization of services using data from primary care EMRs during 2018–2023, as well as describing its geographic distribution.
2. Assessing the impact of COVID-19 on mortality, frailty, functional and cognitive capacity, and quality of life of healthcare users.
3. Understanding and interpreting the social, political, and economic dimensions associated with the use of health services by individuals aged ≥75 years and their family caregivers during the pandemic.

### 2.2 Design/methodology

#### 2.2.1 Study design and setting

Multicentric, mixed models study combining quantitative and qualitative methodologies. The study will be conducted in the primary care setting of 11 of the 19 Spanish administrative regions and will include both insular and peninsular, rural and urban populations, as well as different socioeconomic levels. Three separate studies will be conducted to provide answers to each specific objective, as described below. Integration will be addressed in the methodological level through connecting, to help to understand contextual factors in the quantitative phase or in the selection of participants in the qualitative phase, Integration will also be tackled at the Interpretation and Reporting Level, integrating the findings through narrative in a stage approach, where the results are analyzed and published separately (21).

We have followed the SPIRIT Checklist to address trial protocol and related documents.

**Figure 1:**
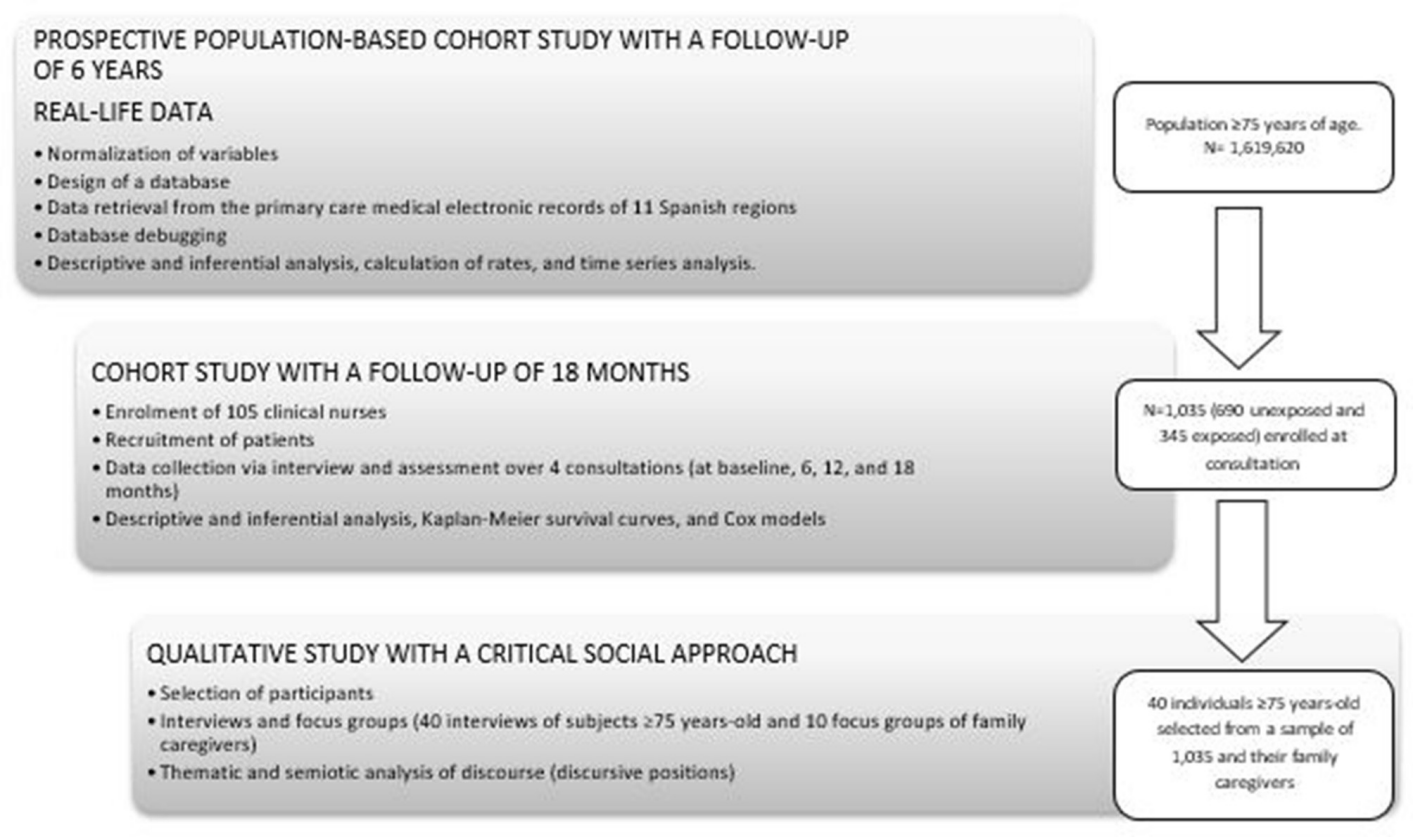
General study design.

The general study design can be found in Figure 1.

#### 2.2.2 Prospective study of an ambispective cohort, with a multipurpose approach, real-life data, and a follow-up of 6 years

##### 2.2.2.1 Population

People aged ≥75 years in January 2018, assigned to any of the health centers of the Spanish National Health System in one of the 11 regions participating in the study. They must have at least one entry in the EMR during 2018, not be institutionalized, and not be mutualists at the time of the cutoff. No sampling is performed, because data will be extracted from the whole population that meets the inclusion criteria.

##### 2.2.2.2 Data source

Primary care EMRs.

##### 2.2.2.3 Variables

Sociodemographic and clinical data; functional, cognitive, and mental status; frailty; social risk; functional patterns; nursing diagnoses, outcomes, and interventions; and use of health services.

##### 2.2.2.4 Data collection

A protocol for standardized variables will be developed based on the Minimum Basic Data Set for Primary Care (MBDS-AP), defined by the Spanish Ministry of Health. Information recorded under real clinical practice conditions between 2018 and 2023 will be extracted from the EMRs. To standardize the variables collected, we used as a reference the consensus document of the Ministry of Health called the Primary Care Clinical Database (BDCAP), which standardizes the measurement instruments and the typology of the variables collected in the electronic medical record throughout the country.

##### 2.2.2.5 Analysis

The study will describe the included population, their care needs, and nursing interventions, all with 95% confidence intervals (CI). The use of health services will be reported as the utilization rate per 1,000 person-year. Incidence rates of new diagnoses and interventions will be calculated per 1,000 person-year with their 95%CI for each follow-up year. The evolution of diagnoses and interventions will be examined using time series analysis. Finally, the geographical distribution of needs will also be described.

#### 2.2.3 Cohort study with a follow-up of 18 months

##### 2.2.3.1 Population

Based on the population of the prospective study, the participants will be ≥75 years of age, with at least one record in the last half of 2019 and another one in the first half of 2022, will not be institutionalized, and must provide informed written consent. Subjects with sensory deficits, cognitive impairment, a Barthel ADL index ≤ 5 points, life expectancy ≤ 9 months, or participants transferred during the recruitment period will be excluded.

##### 2.2.3.2 Sample size

Based on the assumption of a proportional hazards model for survival times, the sample size was estimated to allow the comparison of two curves. The probability of a change in functionality of < 5 points at 18 months was previously estimated to be 47% in the unexposed group (22) and this figure was considered to decrease by at least 10% in exposed patients. Considering a ratio of 2:1 between the sample sizes of the unexposed and exposed groups, for a CI of 95%, a power of 80%, and a dropout rate of 20%, the sample size was calculated to be 1,035 subjects (690 unexposed and 345 exposed). A randomized sampling by quotas for the included health centers in each of the regions will be performed.

##### 2.2.3.3 Recruitment

A total of 105 clinical nurses from the participating centers will be recruited and trained. From the previously selected sample, each nurse will invite 10 candidates from their health center to participate (seven unexposed and three exposed), after ensuring that they meet the inclusion and exclusion criteria.

##### 2.2.3.4 Variables

The exposure variable will be SARS-CoV-2 infection confirmed by diagnostic testing between May 11th of 2020 and the recruitment start date. The outcome variables will be mortality, functionality (Barthel scale), quality of life (EuroQol-5D), mental health (Hamilton depression rating scale), cognitive capacity (Mini-Mental State Examination), and frailty (Frailty index). In addition, sociodemographic (age and gender) and clinical, infection-related, and pandemic-context variables will be recorded. The date of the infection diagnosis and length of hospitalization will also be recorded for the cohort of exposed patients, as well as diagnoses of infection during the follow-up for the cohort of unexposed patients.

##### 2.2.3.5 Data collection and follow-up

Nurses will record the information in the Research Electronic Data Capture application (REDCap). The follow-up will be conducted at the consultation or the patient's home and will comprise a period of 18 months for each participant or until their dropout. Variables will be collected at four time points, namely at the baseline and at 6, 12, and 18 months. To achieve comparability of the data collected, the same data collection notebook was used in all the participating centers, which also included validated questionnaires. In addition, all the professionals who collected data were trained in the use of the application and in the collection process in order to standardize the criteria.

##### 2.2.3.6 Analysis

Population characteristics will be described with their relevant 95% CI. The baseline characteristics will be compared between both groups upon enrolment. Kaplan-Meier survival curves will be generated for the main variables, which will be compared using the log-rank test. Cox models will be generated to study the impact of infection on the outcome variables at 6, 12, and 18 months and to adjust for sociodemographic and clinical characteristics of interest.

#### 2.2.4 Qualitative study with a critical social approach

##### 2.2.4.1 Participants

Clinical nurses will invite to participate subjects ≥75 years of age with a specific profile, who are already part of the cohort study, together with their family caregivers. Caregivers with < 6 months as such at the beginning of the pandemic will be excluded, as well as those with sensory or cognitive deficits. Sample selection will be intentional (based on the study aim) although, depending on the emerging development of the study, it will develop toward a theoretical sampling approach.

The sample size for each of the participating regions has been estimated to be about 40 interviews of individuals ≥75 years of age and the focus groups will include 6–10 family caregivers.

##### 2.2.4.2 Data collection

Data will be obtained from semi-structured interviews of healthcare users ≥75 years old and focus groups of caregivers. In the interviews, there will be the same proportion of men and women, and two age groups, from 75 to 84 years and over 85. In the case of groups, the criterion for homogeneity will consist of being a live-in caregiver or not and the criteria for heterogeneity will be age, gender, and years as a caregiver. The interviews and focus groups will follow a predefined script, which will be revised as the analysis process progresses. The duration of interviews is estimated to be 1 h, and the focus groups will last 2 h. Both will be conducted in an accessible place and will be audio-recorded and transcribed.

In order to compare the results between the different regions, the interview guide and the dimensions to be studied were agreed upon by the coordinating team, experts in qualitative methodology. Both, the interviews and the analysis, will be carried out by this same team, which will act in a coordinated manner and share all the information throughout the process, thus ensuring methodological homogeneity in all areas.

##### 2.2.4.3 Analysis

The analysis will be thematic, complemented by a semiotic analysis, with inductive and deductive elements, identifying units of meaning and assigning codes that will be grouped into categories. An analysis of discursive positions will be carried out, considering the roles that condition the topic. Finally, the discourse will be analyzed as a reflection of ideological positions (inequality and domination). The results will be presented as the participants' verbatims together with the interpretation by the researchers. A reflective diary will be kept for which triangulation of techniques and researchers have been contemplated. Transferability will be ensured by the exhaustive and accurate description of the context, the participants, and their perspectives.

### 2.3 Data monitoring

This study has not considered the need to have a group of independent experts assessing the progress, safety data and critical efficacy endpoints, because it is not a RCT. However, according to the regulations of good practice in research, part of the research team will monitoring risk data (such as adverse events), the research team will often monitor efficacy data, and will occasionally monitor data acquisition to ensure the integrity of the data generated in the study. In subproject 1 the whole process of data collection and standardization of the variables, a pilot will be carried out in each administrative region and before the data analysis, the collection will be validated. In subproject 2, the information registered by the clinical nurses in Research Electronic Data Capture Program (REDCap^®^) will be monitored to detect omissions or errors in the register. Investigators will inform ethical committee of any significant deviations from their approved protocols, and will promptly report all unanticipated and nonserious adverse events immediately (we not expected serious adverse events).

The data sets that will be generated and analyzed during the study, as well as the statistical code, will be available upon reasonable request to the corresponding author.

The complete data management plan is available on DMPonline under registration code ID: 1126580.

### 2.4 Ethical considerations

The coordinated study received approval from the Central Research Committee of Primary Healthcare Management and the Ethics Committee for Drugs Research (CEIm). All the participating regions obtained a favorable response from their relevant ethics committees.

Participants in both the cohort and qualitative studies will be provided with written information about the study and will ask to provide informed consent. For the prospective study of the population-based cohort with database analysis, the relevant committee approved the exemption of the request for informed consent. The study will respect the basic ethical principles of autonomy, welfare, justice, and harmlessness, in accordance with the standards of Good Clinical Practice, the Declaration of Helsinki (Fortaleza, 2013), and the European Convention on Bioethics (Convenio de Oviedo, 1997). The processing, communication, and transfer of data have been made in compliance with local regulations in force (Ley Orgánica 3/2018, de 5 de diciembre, de Protección de Datos Personales y garantía de los derechos digitales) and the rights of data access, rectification, cancellation, and opposition (ARCO) contemplated in that law.

## 3 Discussion

Numerous studies were published over the first months of the pandemic about the effect on different health aspects in different populations. The impact on mortality and mental health has been studied in different countries and health systems with unequal results, which leads to believe that the long-term consequences will also vary among them.

In China, a study conducted on the population over 60 years of age showed an increase in loneliness as an emotional response, anxiety, and insomnia (23). Another study in the same country indicated that the quality of life had not recovered 3 months after hospital discharge of patients admitted for COVID-19 (24). In Norway, Walle-Hansen et al. followed up a cohort of people over 60 years old who were alive 6 months after hospital discharge for COVID-19 and their results pointed to long-term functional impairment (25). In Spain, a study on persons over 70 years of age with a 6-month follow-up showed that infection with COVID-19 predisposes to greater functional impairment (26).

To study the long-term impact of the COVID-19 pandemic on the health condition of people aged ≥75 years and their family caregivers, we designed a mixed methods study protocol. Given the complexity of the question, this work aims to address it by incorporating three methodological approaches. Therefore, the study will not only consider objective aspects of health, quality of life, and utilization of health services by the study population but will also provide the perspectives of patients and caregivers on health services, focusing the attention on patient-centered care.

The analysis of real-life data, obtained from a cohort from 11 Spanish regions representative of the peninsular and insular population, rural and urban setting, will favor the external validity of the results and allow their extrapolation to similar contexts. These data will be of great epidemiological value, as the estimated study population exceeds 1.5 million primary care users and the information will be collected in routine clinical practice, including nursing diagnoses. In addition, the collected data will allow observing the changes in the demand for health resources, occurred during and after the pandemic, based on the information automatically generated by the system (e.g., number and types of consultations). By following up two cohorts over 18 months, this study aims to explore if there have been changes in the aging process of the included population and whether these may be due to the biological impact of the disease, or the situation experienced during the pandemic.

One strength of this study to highlight is the robustness of the outcome variables, which are mostly self-reported outcomes or from EMRs and SNLs registries. The enrolment and follow-up of participants will be conducted by their assigned primary care nurses, which will minimize dropouts and increase the quality of records. In order to avoid selection bias, the cohort of subjects exposed to COVID-19 will only include those individuals diagnosed after May 11th of 2020, as that was the time point at which the diagnosis required confirmatory testing in Spain.

The qualitative methodology uses interviews and focus groups to provide a vision of the reality experienced by the study population. This approach will allow understanding and interpreting the social, political, and economic dimensions associated with the use of health services by patients and caregivers.

### 3.1 Limitations

The quality and homogeneity of data obtained from EMRs can entail a limitation. To overcome this, some of the database sources have been previously validated in epidemiological studies, standardized variables have been selected, and the research team will endeavor to standardize the selected data. A survival bias that underestimates the effect of the disease is another potential limitation inherent to cohort follow-up. For the qualitative fieldwork, the complexity of the studied population may pose difficulties in terms of discourse saturation and narrative quality. This complexity has been taken into consideration by proposing sufficient time slots for the sample selection and data collection processes. For the organization of focus groups, various available schedules will be offered to meet the time needs of the caregivers.

### 3.2 Applicability

We attempt to describe the aging process from a nursing perspective, and to achieve a better understanding of how the pandemic and the measures taken to prevent the spread of the virus affected the health of the population over 75 years and their morb-mortidity. In addition, it will allow us to know the use of services of this population, which will allow us to adjust the size of health services to ensure adequate care for this population. We hope that this information will allow us to adapt health policies in case a similar situation arises in the future. It will also allow us to understand what the needs of these people and their caregivers are, so that we can adapt our health and care policies to them.

In order to promote the results of this study to the scientific community and the general public, it is planned to publish them in open access scientific journals and to present them in different scientific forums. In addition, a web page (https://cuidamos75.com/) has been designed to follow the development of the project and its preliminary results.

On the other hand, the citizen advisory group, in addition to having participated in the design, collaborates in the dissemination strategy of the results to make them more accessible to the lay population.

## 4 Conclusion

The findings of this study could provide the necessary knowledge to build the health and care of the future, set the basis to develop interventions for improving the health condition of people ≥75 years old and their caregivers and to adapt the health system to the new current situations like that created by the COVID-19 pandemic and future challenges facing health and care systems.

## Author contributions

MR-B: Conceptualization, Funding acquisition, Investigation, Methodology, Project administration, Supervision, Writing – original draft, Writing – review & editing. SE-S: Conceptualization, Funding acquisition, Investigation, Methodology, Project administration, Supervision, Writing – review & editing. RS-R: Conceptualization, Investigation, Methodology, Project administration, Supervision, Writing – original draft, Writing – review & editing. AA-E: Writing – review & editing. EA-B: Writing – review & editing. PB-B: Writing – review & editing, Methodology. EC-R: Writing – review & editing. MC-M: Writing – review & editing. MF-J: Writing – review & editing. IF-R: Writing – review & editing. MH-P: Writing – review & editing. CL-H: Writing – original draft, Writing – review & editing. MM-C: Conceptualization, Funding acquisition, Investigation, Methodology, Project administration, Supervision, Writing – review & editing. PO-R: Writing – review & editing. AP-S: Writing – review & editing. AP-M: Conceptualization, Data curation, Funding acquisition, Investigation, Methodology, Project administration, Supervision, Writing – review & editing. ER-R: Writing – original draft, Writing – review & editing. MS-A: Writing – review & editing. JT-A: Writing – review & editing. MV-T: Writing – review & editing. VG-C: Investigation, Supervision, Writing – original draft, Writing – review & editing, Methodology.

## CUIDAMOS+75 Group

Rico, Milagros; Esteban, Silvia; Moreno, Teresa; Abad, Eva; Ariztegui, Ana; Artigues, Eva; Avendaño, Almudena; Bernabeu, Clara; Blanco, Joan; Boixadera, Mireia; Cabeza, Antonio; Cameselle, Candela; Casado, Elvira; Cidoncha, M Ángeles; Company, Consuelo; Cortes, Elisa Belén; Etxebarria, Aitziber; Fabregat, Inmaculada; Feria, Isabel; Fernández, Roser; Galán M, José; García, Marta; Gimeno, Iraida; González, Víctor M; Hernandez, Montserrat; Izaguirre, Eva; López, Yolanda; Losada, Antonio; Lozano, Cristina; Martín, Ángel; Martín, Susana; Más, Marta; Mateo, Ana; Miralles, Jerónima; Moreno, Maribel Noel, Rosa; Oter, Cristina; Otones, Pedro; Padilla, Jorge Rafael; Palmar, Ana M; Pastor, Monica; Pedraz, Azucena; Pisà, Marta; Reina, Gloria; Rich, Manuel; Sánchez, Raquel; Romero, Esperanza M; Ruymán, Pedro; Sarabia, Carmen M; Solé, María; Taltavull, Joana Maria; Vicente, Sergio; Vidal, Clara.
